# Recent Advances in the Synthesis of 2*H*-Pyrans

**DOI:** 10.3390/molecules24162904

**Published:** 2019-08-09

**Authors:** David Tejedor, Samuel Delgado-Hernández, Raquel Diana-Rivero, Abián Díaz-Díaz, Fernando García-Tellado

**Affiliations:** 1 Instituto de Productos Naturales y Agrobiología, Consejo Superior de Investigaciones Científicas, Astrofísico Francisco Sánchez 3, 38206 La Laguna, Tenerife, Spain; 2 Doctoral and Postgraduate School, Universidad de La Laguna, Astrofísico Francisco Sánchez, SN, 38200 La Laguna, Tenerife, Spain

**Keywords:** 2*H*-pyran, heterocycles, synthesis, valence isomerism, 1-oxa-triene, dienone, oxa-electrocyclization, Knoevenagel, propargyl Claisen, cycloisomerization

## Abstract

In this review, we discuss the nature of the different physicochemical factors affecting the valence isomerism between 2*H*-pyrans (2HPs) and 1-oxatrienes, and we describe the most versatile synthetic methods reported in recent literature to access to 2HPs, with the only exception of 2HPs fused to aromatic rings (i.e., 2*H*-chromenes), which are not included in this review.

## 1. Introduction

**Scheme 1:**
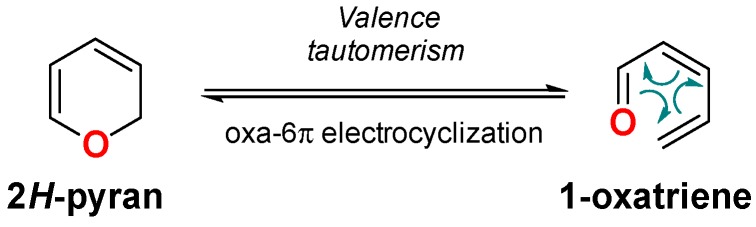
Valence tautomerism of 2*H*-pyrans (2HPs).

**Figure 1:**
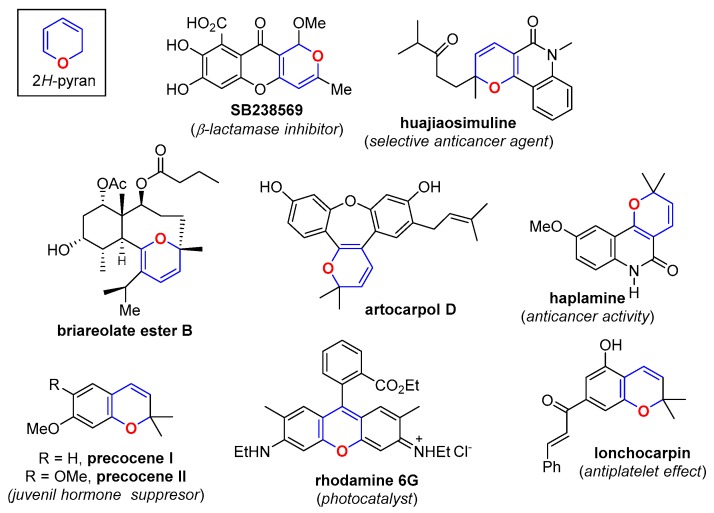
Examples of natural products containing the 2HP motif.

The 2*H*-pyran (2HP) ring constitutes a structural motif present in many natural products (Figure 1) [1] and is a strategic key intermediate in the construction of many of these structures [2,3]. In spite of their importance, the literature of 2HPs is relatively scarce [4,5,6,7,8,9], mainly due to the instability associated with the heterocyclic ring, which makes these heterocycles establish an equilibrium with their opened isomeric forms (Scheme 1). Fusion of a 2HP to an aromatic ring confers stability to these heterocycles. Thus, while simple 2HPs are difficult to synthesize as pure and isolated compounds, many of their benzo derivatives (i.e., 2*H*-chromenes) constitute stable molecules, with a broad spectrum of biological activities and a widespread representation in the higher plants (Figure 1). Because the chemistry and reactivity of 2*H*-chromenes have been already previously revised [1,10,11,12,13,14,15], they will not be included in this review. Instead, we will focus on the recent advances on accessing 2HPs, either as simple and stable monocyclic structures or as part of fused polycyclic structures, excluding the 2*H*-chromene system.

## 2. Dienone/2HP Equilibrium

2HPs are prone to undergo spontaneous valence isomerization [16] to the corresponding 1-oxatrienes through a reversible pericyclic oxa-6π-electrocyclization process (Scheme 1) [17]. This valence tautomerism determines the chemistry of these heterocycles, which commonly exist as a mixture of valence tautomers (isomers) [1]. In this sense, it is important to take into account that because this interconversion is fast in the majority of cases, the method of synthesis used to access these structures does not determine the valence tautomer obtained.

**Scheme 2:**
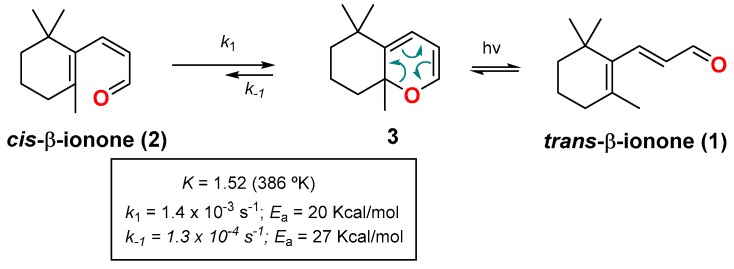
Valence isomerism of *cis*-β-ionone (**2**) and 2HP **3**.

Although this valence isomerization was invoked to explain some enigmatic results found in earlier examples with these molecules, the first clear-cut example of it came from the irradiation of *trans*-β-ionone (**1**) (Scheme 2) [18]. Authors found that the irradiation afforded a mixture of *cis*-β-ionone (**2**) and 2HP **3**, with a value for the equilibrium constant *K* = 4.61 at 327 °K (*K* = 1.52 at 386 °K), and values for *k*_1_ = 1.4 × 10^−3^·s^−1^ and *k*_−1_ = 1.3 × 10^−4^·s^−1^. In addition, measurements at different temperatures gave activation energies (*E*_a_) of 20 Kcal/mol for the *cis*-dienone to 2HP reaction, and 27 Kcal/mol for the reverse process [19].

**Scheme 3:**
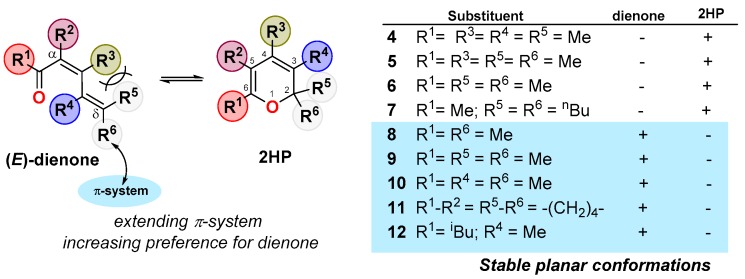
Implications of the *cis*-dienone conformation on the valence isomerism.

Further studies on the conformation of conjugated dienones allowed the establishment of some general patterns for these dienone/2HP equilibria (Scheme 3) [20]. It was observed that steric destabilization of the dienones shifted the equilibria toward the 2HPs. This was the case for tetrasubstituted dienones **4** and **5**, which fully isomerized to the corresponding 2HPs. On the other hand, simpler dienones **8**–**12**, which could adopt a stable planar conformation, existed in the opened form. Likewise, trisubstituted dienones **6**–**7**, which are representative examples of dienones featuring non-stable planar conformations, preferred their closed forms. Along with these results, the authors also observed that the substitution of a δ-alkyl substituent (R^5^ or R^6^) by a substituent able to extend the conjugation of the π-system (e.g, vinyl group) favored the dienone form (Scheme 3). A main conclusion from these and other studies [20,21] is that the existence of the 2HP form depends primarily on the steric destabilization of the dienone rather than on its specific substitution pattern. Thus, the design of stable 2HPs must include, among other structural/electronic criteria, enough steric destabilization on the dienone to penalize the valence isomerization.

**Scheme 4:**
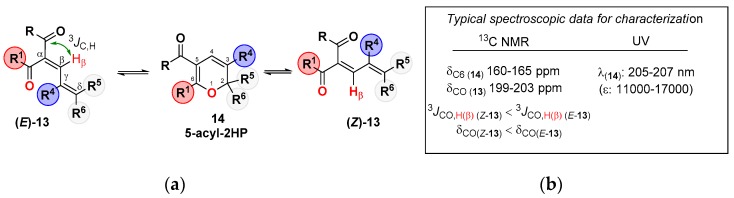
(**a**) Valence tautomerism of 5-acyl-2HPs **14**. (**b**) Spectroscopic characteristic of tautomers.

**Scheme 5:**
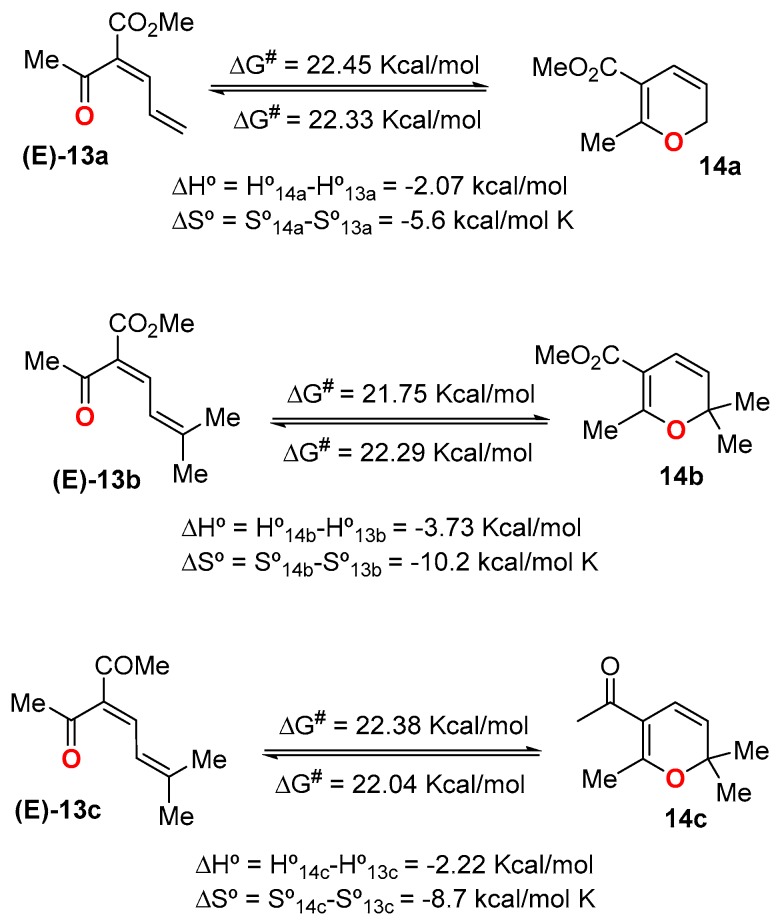
Thermodynamic data for **13a**–**c**/**14a**–**c** valence isomerization.

More recently, Krasnaya et al. carried out a systematic investigation on the influence of substituents and solvents on the valence isomerization of trisubstituted α-acyl-dienones **13** (Scheme 4). In this study, the authors quantified the equilibrium compositions of 26 differently substituted α-acyl-dienones **13** (Table 1), and determined the thermodynamic and activation parameters for some of these equilibria (Scheme 5) [22].

Table 1 summarizes the earlier observed importance of structural effects on the valence equilibrium, and it confirms some general patterns:

1. The successive substitution at position C_2_ in the ring (C_δ_ on dienone) leads to an increase in the content of 2HP (steric strain on the dienone) (compare entries 1–3 and 7–8).
2. The elongation of the conjugated system results in an increase in the content of dienone (resonance delocalization) (compare entries 15, 25 and 17, 26).
3. Substitution at the C_2_-position of the ring (C_δ_ on dienone) with two methyl groups strongly shifts the equilibrium toward the 2HP (entries 17, 19). In this case, it is possible to observe only the 2HP (compare entries 7, 17 with 11, 19).
4. Aprotic polar solvent shifts the equilibrium toward the formation of the dienone.
5. Although the electronic effect of the acyl group at the C_5_-position of the ring (C_α_ in the dienone) is masked in Table 1, other studies have shown that the presence of an electron-withdrawing substituent(s) at the ring, preferentially at this C_5_-position, favors the 2HP [23,24,25]. Table 1 shows that although this effect could be operative in α-acyl-dienones **13**, it can be completely surpassed by other structural/electronic effects (Table 1, entries 11–14).

With regard to thermodynamic parameters of some of these equilibria (Scheme 5), Krasnaya et al. found that, in all cases, the enthalpies of the α-acyl-dienones **13** were appreciably higher than those of 5-acyl-2HPs **14**, which is in full agreement with the observed increase in the dienone content with the increase in temperature. As should be expected, the entropy contents were also higher for the closed structures. In all the investigated cases, ΔG^#^ values were on the order of 21.88 Kcal/mol to 22.86 Kcal/mol.

**Scheme 6:**
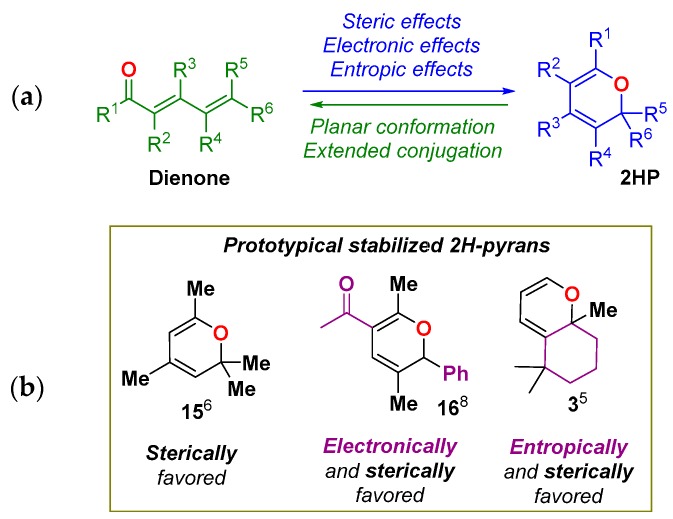
(**a**) Summary of parameters affecting the valence isomerization. (**b**) Prototypical examples of stable 2HPs.

Finally, other structural factors, such as annulation, also favor the 2HP form. It has been well established that annulation favors the closed form by restricting conformational freedom (entropic trap), and it has been used as a design element in the synthesis of stable 2HPs [26,27]. Scheme 6 graphically summarizes the main conclusions of these studies. Structures **3**, **15, 16** represent prototypical examples of room temperature stable 2HPs.

## 3. Synthesis of the 2HP Core

**Scheme 7:**
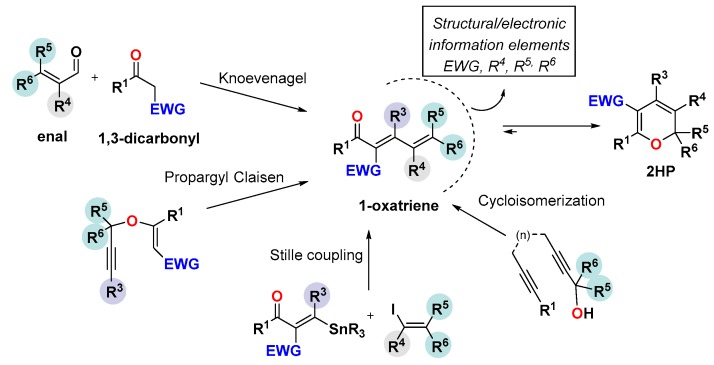
Synthesis of 1-oxatriene core incorporating structural/electronic information.

The most common route for synthesizing these heterocycles relies on the oxa-6π-electrocyclization of dienones, the so-called 1-oxatriene pathway [28]. As already discussed in the previous section, this methodology requires endowing the 1-oxatriene unit with structural or electronic information, or both, to shift the valence equilibrium toward the 2HP form (Scheme 7). Thus, different synthetic pathways to the 1-oxatriene core have been successfully explored, involving, among others, the classic Knoevenagel condensation between active methylene compounds and α, β-unsaturated aldehydes (enals), Claisen rearrangements of propargyl vinyl ethers, Stille coupling of vinyl stannanes and vinyl iodides, and cycloisomerization of dienols (Scheme 7).

### 3.1. The Knoevenagel/Electrocyclization Protocol

**Scheme 8:**
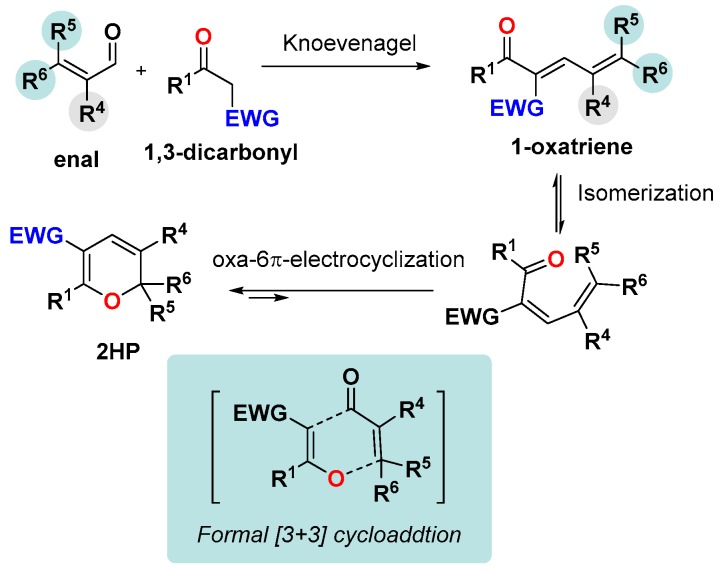
Knoevenagel/electrocyclization strategy.

The Knoevenagel condensation constitutes the most common access to 1-oxatrienes, and most generally involves the reaction of an enal with a 1,3-dicarbonyl compound [29]. The sequential performance of the Knoevenagel condensation and the electrocyclization reaction generates 2HPs (Scheme 8). From a synthetic point of view, the whole tandem process can be considered a formal [3 + 3] cycloaddition [2,28]. There is a plethora of examples of this strategy in the literature, mainly in the field of total synthesis of natural products. In this review, we will pay attention exclusively to established synthetic methods that allow or have allowed general access to these heterocycles. Specific cases utilized to access a particular structure or a specific natural product will not be covered. We refer the reader to the excellent published reviews covering this issue [2,3].

**Scheme 9:**
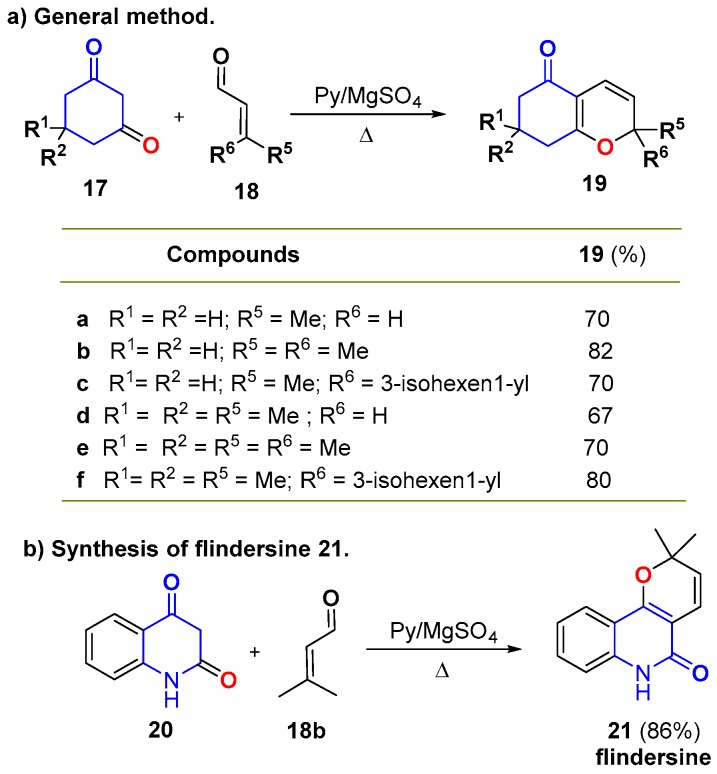
Knoevenagel/electrocyclization: (**a**) synthesis of annellated 2HPs **19** and (**b**) synthesis of flindersine (**21**).

Fusion to a ring favors the electrocyclization of the 1-oxatriene intermediate, and it has been used as a steering element to synthesize stable 2HPs. As an earlier example, the pyridine-mediated condensation of different cyclic 1,3-dicarbonyl compounds **17** and functionalized enals **18** generated the stable bicyclic 2HPs **19** in good yields (Scheme 9a) [30]. Therefore, the double substitution at the terminal position of the enal also contributed to the global stability of 2HPs **19**. Using this methodology, the same authors synthesized the alkaloid flindersine (**21**) in 86% yield and in just one synthetic step (Scheme 9b).

**Scheme 10:**
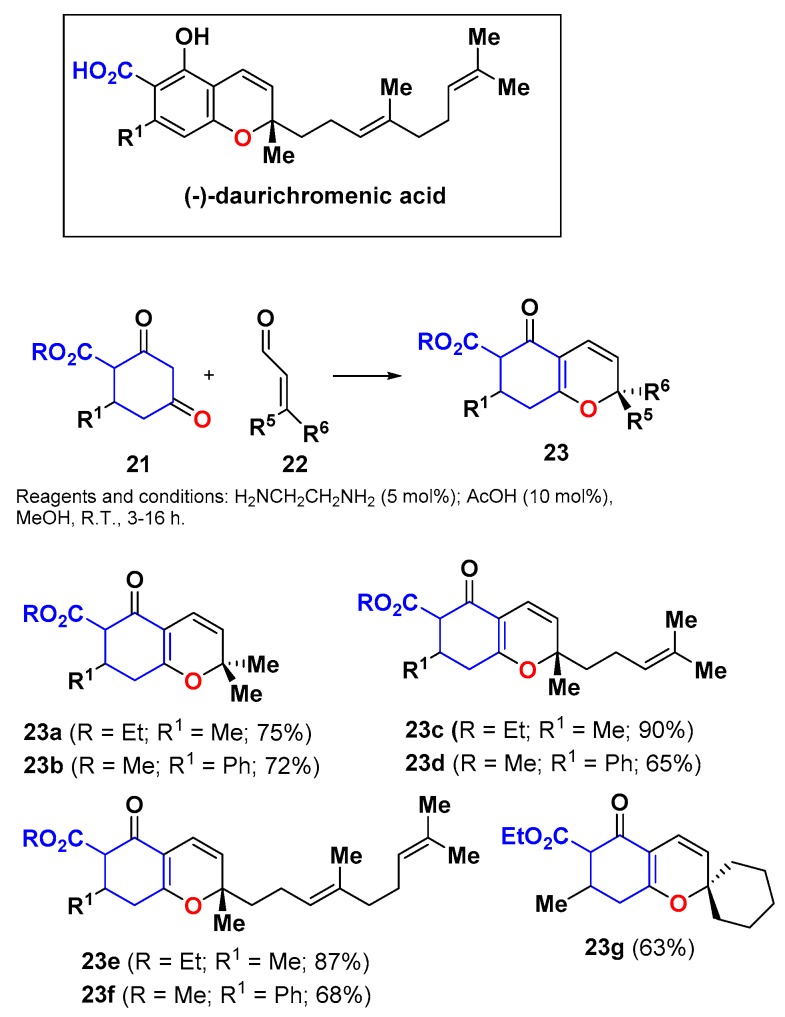
Synthesis of 2HPs **23** by iminium-mediated Knoevenagel condensation (IMKC) of cyclohexa-1,3-dione **21** and enals **22**.

**Scheme 11:**
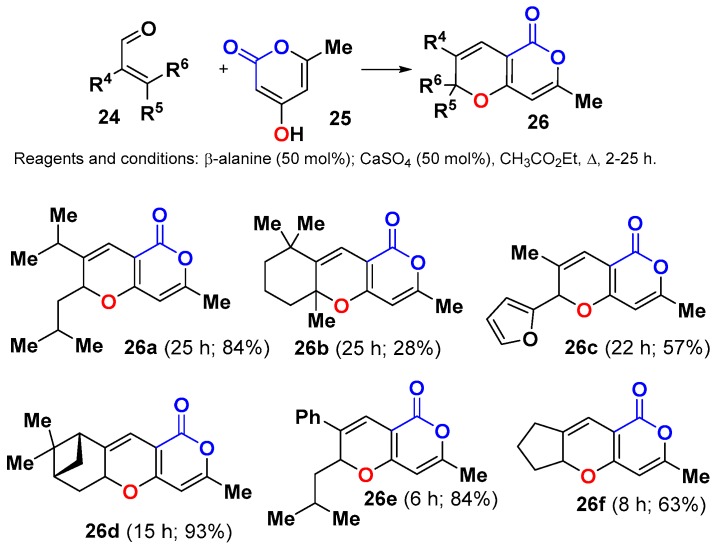
Construction of a small library of annulated 2HPs **26** by the IMKC of 4-hydroxypyranone **25** and functionalized enals **24**. A selection of library members is shown.

The iminium-mediated Knoevenagel condensation (IMKC) [31,32] has been currently used to condense 1,3-dicarbonyl (active methylene) compounds with 2-alkenyliminiums (activated enals), and it constitutes a very versatile route to 1-oxatrienes [2,33]. The chemical outcome of the reaction is that of a formal [3 + 3] cycloaddition between enols and enals (see Scheme 8). The reaction is productive when functionalized cyclohexane-1,3-diones (e.g., **21**) (Scheme 10) or 4-hydroxypyrones **25** (Scheme 11) are used as the active methylene compounds in the process. In this manner, 2HPs **23a**–**g** were synthesized from the functionalized cyclohexa-1,3-dione **21** and different functionalized enals **22** (Scheme 10) [34]. These 2HPs were used as key intermediates in the total synthesis of (−)-daurichromenic acid and analogues. The use of Lewis [35] or Brønsted [36] acids, In^3+^ [37], or iodine [38] as catalysts resulted complementary to the iminium formation and afforded similar reaction outcomes.

This methodology is well suited for use in diversity oriented synthesis programs [39], as long as the structural control elements are incorporated into the library design. Thus, a small and structurally varied library of 2HPs **26** was constructed using the β-alanine-mediated IMKC between 4-hydroxypyranone **25** and different enals **24** (Scheme 11) [40]. In vitro studies of antiproliferative/cytotoxic activity with human SH-SY5Y neuroblastoma cells showed IC_50_ values ranging from 6.7 to >200 μM. 2HP **26a** exhibited the highest cytotoxicity to the neuroblastome cells and necrotic effects on the human IPC melanoma cells.

**Scheme 12:**
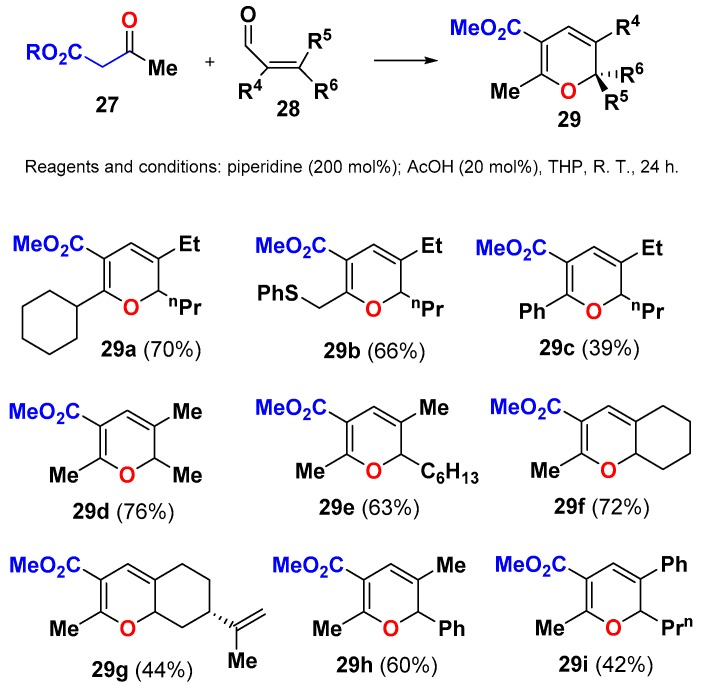
Synthesis of 2,3,6-trisubstituted 2HPs **29** by IMKC of methyl acetoacetate **27** and enals **28**.

Although the use of cyclic 1,3-dienones has been beneficial for the synthesis and stability of the resulting 2HPs, it is not a mandatory requirement, and acyclic active methylene compounds, such as methyl acetoacetate **27**, have been successfully condensed with 2-alkyl-2-enals **28** to deliver the corresponding stable 2,3,6-trisubstituted 2HPs **29** (Scheme 12) [41].

**Scheme 13:**
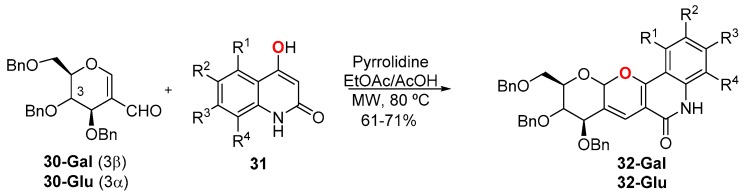
Carbohydrate fused pyrano[3,2-c]quinolone library.

Pyrano[3,2-c]quinolone is a core structural motif in alkaloids and is endowed with important pharmacological and therapeutic activities. As part of a research program aimed at developing efficient synthesis of natural product-like small molecules, a small 23-membered library focused on carbohydrate fused pyrano[3,2-c]quinolone structures **32** was synthesized and subjected to antiproliferative activity studies (Scheme 13) [42]. The library was synthesized using the microwave assisted pyrrolidine-AcOH catalyzed IMKC of formyl galactal (30-Gal) and formyl glucal (30-Glu) with 4-hydroxyquinolones **31**, and although the electron donating or electron withdrawing character of groups R^1^, R^2^, R^3^, and R^4^ of 4-hydroxyquinolones significantly affected neither the yield nor the reaction completion time, the best yields were obtained when unsubstituted **31** was used (70% with 30-Gal and 71% with 30-Glu). The other combinations gave yields ranging from 62 to 69%.

**Scheme 14:**
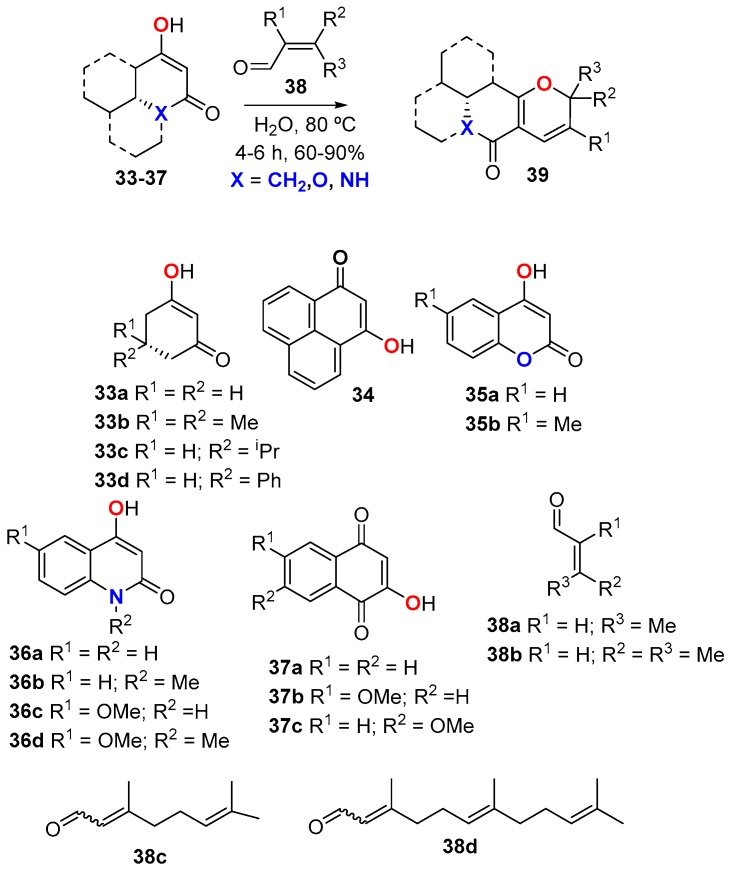
Knoevenagel/electrocyclization in water.

The Knoevenagel/electrocyclization strategy is suitable to be performed in water (Scheme 14) [43]. This methodology was applied to the synthesis of biologically interesting 2HPs of general structure **39**, comprising pyranocoumarin, pyranoquinolinone, and pyranonaphthoquinone derivatives along with selected natural and non-natural products (X = CH_2_, O, NH). The reactions were performed by mixing the 1,3-dicarbonyl compound **33**–**37** with enal **38** in water at 80 °C for 4–6 h. Although authors did not specify the physical conditions of these reactions, the high hydrophobicity of the reactants suggested that these reactions were carried out as aqueous suspensions (the so-called “on water” conditions [44], rather than as homogeneous solutions. Solvent-free protocols have been also described for the IMKC reaction [45,46].

### 3.2. From Other Heterocycles

**Scheme 15:**
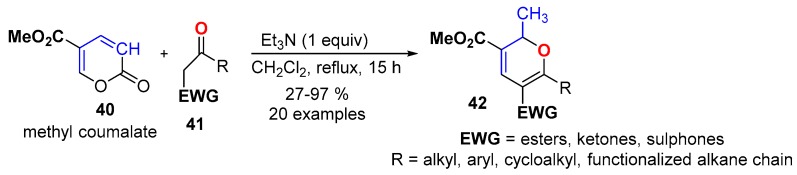
Domino synthesis of tetrasubstituted 2HPs **42** from methyl coumalate **40**.

The condensation of methyl coumalate (**40**) with a wide range of active methylene compounds **41** has been implemented to access an extensive series of 2,3,5,6-tetrasubstituted 2HPs **42** (Scheme 15) [47]. The reaction involved a domino 1,6-Michael/6π-electrocyclic ring opening/[1,5]-*H* transfer/(decarboxylation)/6π-electrocyclization reaction. The methyl substituent allocated at C_2_ position of the 2HP ring corresponds to the α-methine group alpha to the lactone in the coumalate ring (highlighted as CH in Scheme 15).

**Scheme 16:**
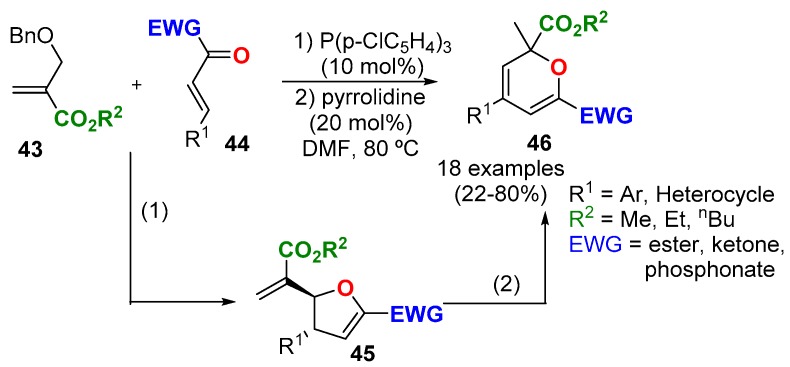
Synthesis of tetrasubstituted 2HPs **46** from 4,5-dihydrofurane **45**.

A one pot synthesis of 2,2,4,6-tetrasubstituted 2HPs **46** has been developed using Bayllis–Hillman carbonates **43** and β,γ-unsaturated α-oxo-esters **44** (Scheme 16) [48]. The one-pot reaction involved a phosphine-catalyzed condensation of **43** and **44** to give intermediate 4,5-dihydrofuran **45**, which, in the presence of pyrrolidine and heat, rearranged to the 2HP **46**. Authors gave a tentative mechanism for this pyrrolidine-catalyzed rearrangement. All the examples incorporated an aromatic (heterocyclic) substituent at R^1^, but the authors do not explain if this was a mandatory property of this substituent.

### 3.3. From Allenolates

**Scheme 17:**
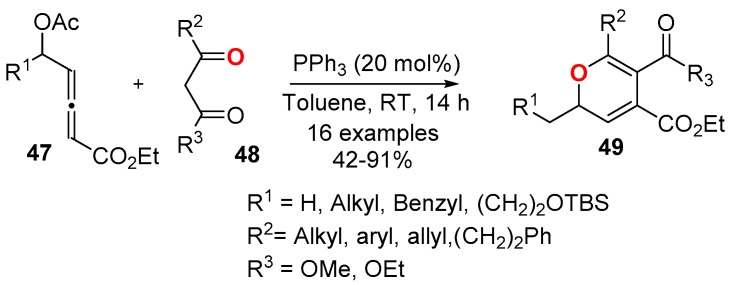
PPh_3_-catalyzed synthesis of 2,4,5,6-tetrasubstituted 2HPs **49** from ethyl 5-acetoxypent-2,3-dienoate **47** and 1,3-dicarbonyl compounds **48**.

Stable 2,4,5,6-tetrasubstituted 2HPs **49** have been synthesized by the phosphine-catalyzed [3 + 3] annulation of ethyl 5-acetoxypenta-2,3-dienoate **47** and 1,3-dicarbonyl compounds **48** (Scheme 17) [49]. The scope of the reaction was wide, tolerating a good variety of the substituents. The presence of the ester group at the C_5_ position of the ring was fundamental for the stability of the 2HP **49**.

### 3.4. From Alkynes 

#### 3.4.1. From Propargyl Vinyl Ethers

**Scheme 18:**
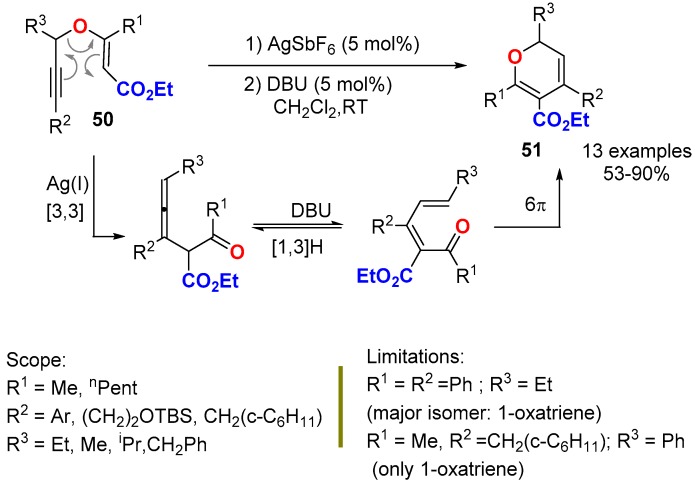
One pot synthesis of 2,3,4,6-tetrasubstituted 2HPs **51** from propargyl vinyl ethers **50**.

Propargyl vinyl ethers **50** have been successfully rearranged into stable 2,4,5,6-tetrasubstituted 2HPs **51** through a one pot procedure involving a Ag(I)-catalyzed propargyl Claisen rearrangement followed by a tandem DBU-catalyzed isomerization/6π-oxa-electrocyclization reaction (Scheme 18) [50]. The protocol used secondary propargyl vinyl ethers (they bear only one substituent at the propargylic position; R^3^), and it required the installation of an ester group at the C_5_ position of the ring and substitution at the C_6_ position to give stability to the monocyclic 2HP **51**.

**Scheme 19:**
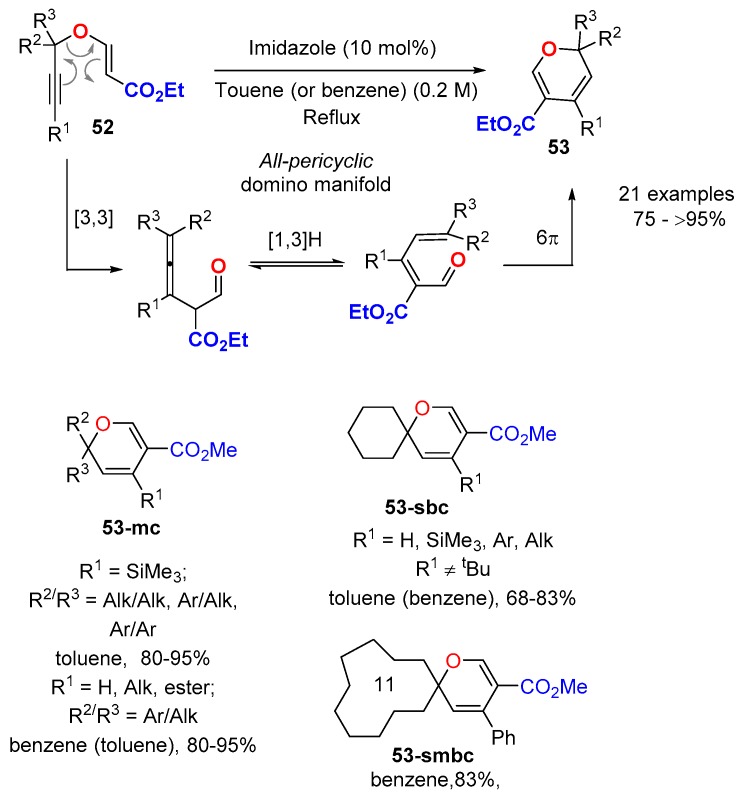
All pericyclic domino synthesis of 2,2,4,5-tetrasubstituted 2HPs **53** from propargyl vinyl ethers **52**.

More recently, a metal-free domino strategy has been developed for the synthesis of 2,2,4,5-tetrasubstituted 2HPs **53** from propargyl vinyl esters **52** (Scheme 19) [51,52,53]. The strategy made use of an imidazole-catalyzed all-pericyclic domino manifold entailing a sequential propargyl Claisen rearrangement/[1,3]-*H* shift/oxa-6π-electrocyclization set of reactions. Again, the presence of an ester functionality at the position C_5_ of the ring was mandatory to stabilize the final 2HP **53**. The double substitution at C_2_ favored the 2HP formation (steric information) and offered a wide range of optional substitution patterns at the ring (Alk/Alk, Ar/Alk, Ar/Ar). The protocol delivered 2HP structures endowed with different topologies, including monocycles (**53**-**mc**), 2,2-spiro-bicycles (**53**-**sbc**), and 2,2-spiro-macrobicycles (**53**-**smbc**). The main limitation arose from the presence of a ^t^Bu substituent at the alkyne position (R^1^): In this case, the reaction followed a different pathway through a sequential [1,7]-*H* shift/6π-electrocyclization/MeOH elimination set of reactions [54].

**Scheme 20:**
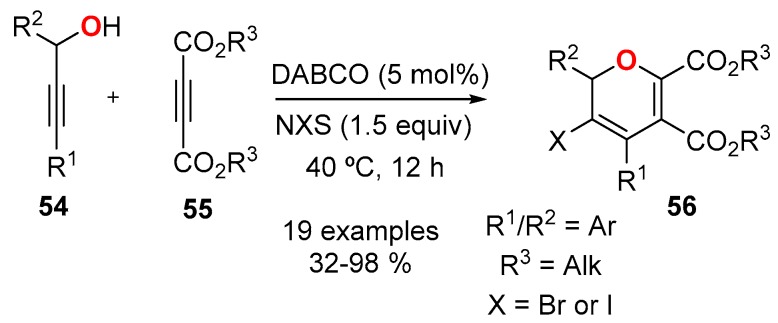
Synthesis of 2,3,4,5,6-pentasubstituted 2HP **56** from propargyl alcohols **54** and dialkyl acetelynedicarboxylates **55**.

An alternative protocol using propargyl alcohols **54** and alkyl ethylendicarboxylates **55** has been developed (Scheme 20) [24,55]. The protocol generated 2,3,4,5,6-pentasubstituted 2HPs **56**, incorporating two identical ester functionalities at C_5_ and C_6_, and a halogen atom at C_3_. The protocol used DABCO as the catalyst and N-iodosuccinimide (NIS) or N-bromosuccinimide (NBS) as the halogenation agent to generate 2HPs **56** in moderate to good yields. In all the conditions explored in Scheme 20, the substituents at the propargyl alcohol were aromatics (R^1^/R^2^ = Ar). The authors did not specify if this was a limitation to the procedure, or was just an inconvenient choice of starting materials.

#### 3.4.2. From Diynes

**Scheme 21:**
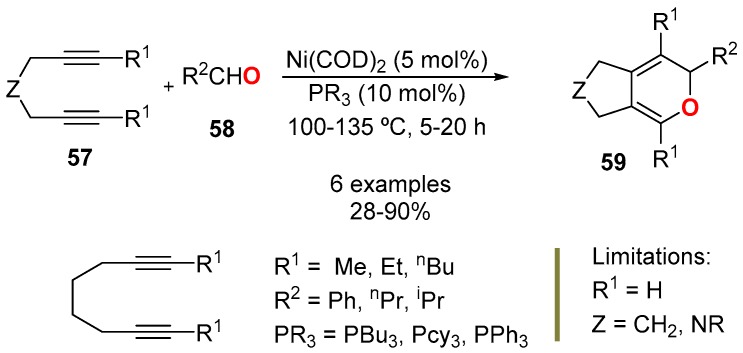
Ni(0)-catalyzed synthesis of bicyclic 2HP **59** from diynes **57** aldehydes **58**.

The Ni(0)-catalyzed cycloaddition of diynes **57** and aldehydes **58** has been explored in the construction of bicyclic 2HPs **59** (Scheme 21) [26,56]. Authors found that the structure of the diyne **57**, mainly the substitution at the terminal positions (R^1^ ≠ H), and the length of the chain connecting the alkyne units, exerted a great influence on the bicyclic 2HP formation reaction. The worst yield was observed when acetaldehyde was used as the aldehyde (28%), whereas the best was observed with *n*-butanal (90%).

**Scheme 22:**
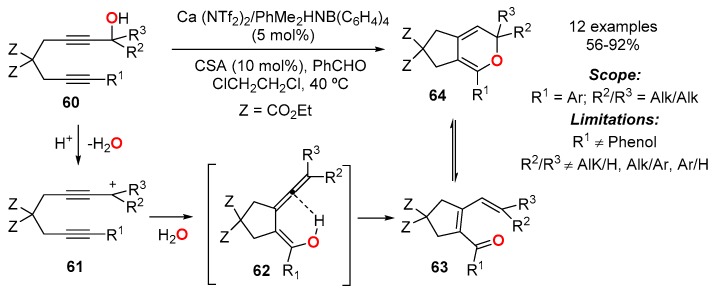
Ca^2+^/H^+^-catalyzed synthesis of bicyclic 2HP **64** from diynols **60**.

A transition metal-free, cycloisomerization of diynols **60** to generate bicyclic 2HPs **61** has been reported (Scheme 22) [26]. The reaction was catalyzed by a cooperative catalytic system entailing Ca^2+^ catalyst (5 mol%) and camphorsulphonic acid (10 mol%), in the presence of benzaldehyde as a mild Lewis basic electron donor. The reaction was carried out without exclusion of air and moisture, and it tolerated a wide range of functionalities on the electron rich 2HP ring. The main limitations arose from substituents R^2^/R^3^ at the propargylic terminal position, which only allowed the alkyl/alkyl combination, and from R^1^, which had to be aromatic. The only limitation for the aromatic substituent at R^1^ was the presence of a free hydroxyl group at the *ortho* position of the ring. As long these restrictions were kept, excellent yields of 2HPs were obtained. The mechanistic proposal involved the formation of a propargylic tertiary cation **61**, which afforded the cyclic 1-oxa-2,4,5-triene intermediate **62**, which isomerized to **63** and rearranged into 2HP **64**.

#### 3.4.3. From Alkenes: Tandem Stille-Oxa-Electrocyclization Reaction

**Scheme 23:**
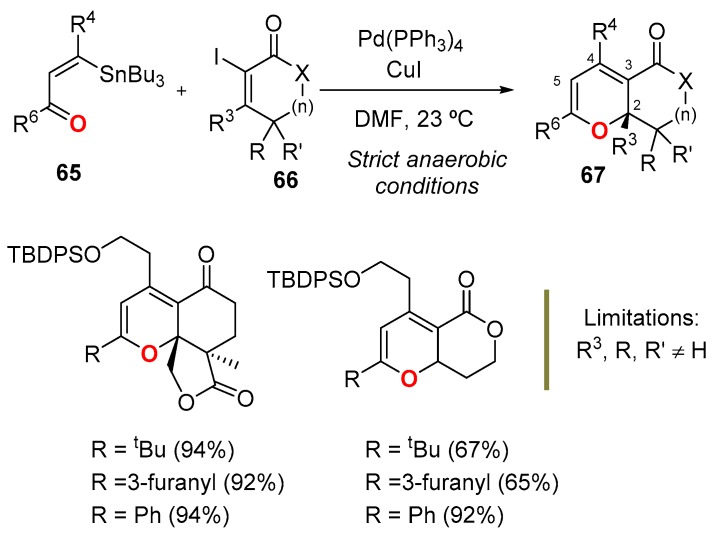
Stille-oxa-electrocyclization strategy to access bicyclic 2HP **67**.

Highly substituted bicyclic 2HPs **67** have been synthesized by a palladium-catalyzed tandem Stille-oxa-electrocyclization reaction between vinyl stannanes **65** and vinyl iodides **66** (Scheme 23) [57,58]. The strategy was a convergent alternative to the known methods for constructing similar bicyclic 2HPs, and it has been used in the total synthesis of natural products [59,60,61]. Although it required the prior stereoselective construction of both vinyl derivatives, the strategy had several advantages: It was convergent, highly diastereoselective, and required mild reaction conditions with low catalyst loadings (5 mol%). In the pattern of construction depicted in Scheme 23, the main restriction came from the nature of the vinyl iodide **66**, which had to have substituents at the vinyl (R^3^ ≠ H) and allylic positions (R/R’ ≠ H) to stabilize the 2HP ring form by steric destabilization of the 1-oxatriene form.

## 4. Summary and Conclusions

We have discussed the structural and electronic factors controlling the valence isomerism of 2HPs and how they can be harnessed to design effective synthesis of 2HPs. The most common routes to access these heterocycles relies on the 6π-electrocyclization of the corresponding 1-oxatriene isomers; thus, the synthetic challenge translates into the synthesis of the 1-oxatriene precursor. We have gathered the most transited routes to these species, including the proper Knoevenagel reaction, the tandem propargyl Claisen rearrangement/[1,3]-*H* shift reactions hosted by propargyl vinyl ethers, the cycloisomerization of diynes, and the Stille coupling of vinyl iodides and vinyl stannanes. From the large number of methods reported in the literature to access these heterocycles, we have selected only those able to generate stable rings with a convenient amount of structural/functional diversity. We hope that this review has filled the existing gap in literature regarding the reactivity and synthesis of these heterocycles, and that it finds use in future applications of these heterocycles.

## Figures and Tables

**Table 1:** α-Acyl-dienones **13** and their equilibrium isomeric compositions.^a^

Entry	R	R^1^	R^4^	R^5^	R^6^	(*E*)-13	2HP 14	(*Z*)-13
1	EtO	Me	H	H	Me	30	30	40
2	EtO	Me	H	H	H	45	30	25
3	EtO	Me	H	Me	Me	17	68	15
4	MeO	Me	H	H	H	43	37	30
5	MeO	Me	H	H	Me	40	40	20
6	MeO	Me	H	Me	Me	26	62	12
7	Me	Me	H	H	Me	72	28	-
8	Me	Me	H	Me	Me	64	36	-
9	*t*-BuO	Me	H	Me	Me	18	17	65
10	EtO	Ar^b^	H	Me	Me	84	9	7
11	EtO	Ph	H	H	Me	67	-	33
12	EtO	Ph	H	Me	Me	86	-	14
13	EtO	Me	H	H	Ph	60	-	40
14	Me	Me	H	H	Ph	100	-	-
15	MeO	Me	Me	Me	Me	-	83	17
16	MeO	Me	Me	H	Ph	-	100	-
17	Me	Me	Me	Me	Me	-	100	-
18	Me	Me	Me	H	Ph	-	100	-
19	EtO	Ph	Me	Me	Me	-	100	-
20	MeO	Me	H	H	c-C_6_H_11_	30	23	47
21	MeO	Me	Me	H	c-C_6_H_11_	-	100	-
22	MeO	Me	H	–(CH_2_)_5_–		16	67	17
23	MeO	Me	Me	–(CH_2_)_5_–		-	100	-
24	MeO	Me	H	–(CH_2_)_4_–		47	31	22
25	MeO	Me	Me	H	HC=CMe_2_	75	-	25
26	Me	Me	Me	H	HC=CMe_2_	100^c^	-	-

^a^ The composition was determined by ^1^H-NMR in CDCl_3_ at 30 °C. ^b^ Ar = *p*-nitrophenyl. ^c^ The *E* and *Z* are topomers.

## References

[1] Fravel B.W., Katritzky A.R., Ramsdem C.A., Scriven E.F.V., Taylor R.J.K. (2008). Pyrans and their Benzo Derivatives: Applications. Comprehensive Heterocyclic Chemistry III.

[2] Hsung R.P., Kurdyumov A.V., Sydorenko N. (2005). A formal [3 + 3] cycloaddition approach to Natural-Product synthesis. Eur. J. Org. Chem..

[3] Beaudry C.M., Malerich J.P., Trauner D. (2005). Biosynthetic and biomimetic electrocyclizations. Chem. Rev..

[4] Drygina O.V., Garnovskii A.D., Kazantsev A.V. (1985). 2*H*-pyrans (review). Chem. Heterocycl. Compd..

[5] Brimble M.A., Gibson J.S., Sperry J., Katritzky A.R., Ramsdem C.A., Scriven E.F.V., Taylor R.J.K. (2008). Pyrans and their Benzo Derivatives: Synthesis. Comprehensive Heterocycles Chemistry III.

[6] Hepworth J.D., Gabbutt C.D., Heron B.M., Katritzky A.R., Rees C.W., Scriven R.J.K. (1996). Pyrans and their Benzo Derivatives: Synthesis. Comprehensive Heterocycles Chemistry II.

[7] Hepworth J.D., Katritzky A.R., Rees C.W. (1984). Pyrans and Fused Pyrans: (iii) Synthesis and Applications. Comprehensive Heterocycles Chemistry.

[8] Hepworth J.D., Heron B.M., Gribble G.W., Joule J.A. (2009). Six-membered ring systems: With O and/or S atoms. Progress in Heterocyclic Chemistry.

[9] Kuthan J., Šebek P., Böhm S. (1995). New Developments in the Chemistry of Pyrans. Adv. Heterocyclic. Chem..

[10] Pratap R., Ram V.J. (2014). Natural and Synthetic Chromenes, Fused Chromenes, and Versatility of Dihydrobenzo[*h*]chromenes in Organic Synthesis. Chem. Rev..

[11] Majumdar N., Paul N.P., Mandal S., de Bruin B., Wulff W.D. (2015). Catalytic Synthesis of 2*H*-Chromenes. ACS Catal..

[12] Ellis G.P., Lockhart I.M., Ellis G.P. (2009). The Chemistry of Heterocyclic Compounds: Chromenes, Chromanones, and Chromones.

[13] Ellis G.P., Katritzky A.R., Rees C.W. (1984). Pyrans and fused pyrans: (ii) reactivity. Comprehensive Heterocycles Chemistry.

[14] Schweizer E.E., Meeder-Nycz O., Ellis G.P. (1977). Chromenes, Chromanes, Chromones.

[15] Hepworth J.D., Heron B.M., Gribble G.W., Joule J.A. (2005). Synthesis and photochromic properties of naphthopyrans. Progress in Heterocyclic Chemistry.

[16] Vogel E. (1963). Valence Isomerizations in compounds with strained rings. Angew. Chem. Int. Ed. Engl..

[17] Rodriguez-Otero J. (1999). Study of the electrocyclization of (*Z*)-hexa-1,3,5-triene and its heterosubstituted analogues based on ab initio and DFT calculations. J. Org. Chem..

[18] Marvell E.N., Caple G., Gosink T.A., Zimmer G. (1966). Valence isomerization of a *cis*-dienone to an α-pyran. J. Am. Chem. Soc..

[19] Marvell E.N., Gosink T.A. (1972). Valence isomerization of 2,4,6-trimethyl-2*H*-pyran. J. Org. Chem..

[20] Lillya C.P., Kluge A.F. (1971). Molecular spectra and conformations of conjugated dienones. J. Org. Chem..

[21] Gosink T.A. (1974). Valence isomers. Substituent effects on the equilibrium between 2*H*-pyrans and cis dienones. J. Org. Chem..

[22] Krasnaya Z.A. (1999). Dienone ⇄ *2H*-pyran valence isomerization. Chem. Heterocycl. Comp..

[23] Adams R.D., Chen L. (1994). Coupling of alkynes to carbon monoxide at a dimanganese center. A new route to carboxylate-functionalized pyrans. J. Am. Chem. Soc..

[24] Fan M., Yan Z., Liu W., Liang Y. (2005). DABCO-catalyzed reaction of α-halo carbonyl compounds with dimethyl acetylenedicarboxylate: A novel method for the preparation of polysubstituted furans and highly functionalized 2*H*-pyrans. J. Org. Chem..

[25] Qing F.L., Gao W.Z. (2000). The first synthesis of 4-trifluoromethyl-2*H*-pyrans by palladium-catalyzed cyclization of (*E*)-3-alkynyl-3-trifluoromethyl allylic alcohols. Tetrahedron Lett..

[26] Rauser M., Schroeder S., Niggemann M. (2015). Cooperative catalysis: Calcium and camphorsulfonic acid catalyzed cycloisomerization of diynols. Chem. Eur. J..

[27] Tsuda T., Kiyoi T., Miyane T., Saegusa T. (1988). Nickel(0)-catalyzed reaction of diynes with aldehydes. J. Am. Chem. Soc..

[28] Voskressensky L.G., Festa A.A., Varlamov A.V. (2014). Domino reactions based on Knoevenagel condensation in the synthesis of heterocyclic compounds. Recent advances. Tetrahedron.

[29] Tietze L.F., Beifuss U., Trost B.M. (1992). The Knoevenagel reaction. Comprehensive Organic Synthesis.

[30] de Groot A., Jansen B.J.M. (1975). A simple synthesis of 2*H*-pyrans; a one-step synthesis of flindersine. Tetrahedron Lett..

[31] Lelais G., MacMillan D.W.C. (2006). Modern Strategies in organic catalysis: The advent and development of iminium activation. Aldrichimica Acta.

[32] Erkkila A., Majander I., Pihko P.M. (2007). Iminium catalysis. Chem. Rev..

[33] Shen H.C., Wang J., Cole K.P., McLaughlin M.J., Morgan C.D., Douglas C.J., Hsung R.P., Coverdale H.A., Gerasyuto A.I., Hahn J.M. (2003). A formal [3 + 3] cycloaddition reaction. improved reactivity using α,β-unsaturated iminium salts and evidence for reversibility of 6π-electron electrocyclic ring closure of 1-oxatrienes. J. Org. Chem..

[34] Hu H., Harrison T.J., Wilson P.D. (2004). A modular and concise total synthesis of (±)-daurichromenic acid and analogues. J. Org. Chem..

[35] Kurdyumov A.V., Lin N., Hsung R.P., Gullickson G.C., Cole K.P., Sydorenko N., Swidorski J.J. (2006). A Lewis acid-catalyzed formal [3 + 3] cycloaddition of α,β-unsaturated aldehydes with 4-hydroxy-2-pyrone, diketones, and vinylogous esters. Org. Lett..

[36] Hubert C., Moreau J., Batany J., Duboc A., Hurvois J.P., Renaud J.L. (2008). Brønsted acid-catalyzed synthesis of pyrans via a formal [3 + 3] cycloaddition. Adv. Synth. Catal..

[37] Lee Y.R., Kim D.H., Shim J.J., Kim S.K., Park J.H., Cha J.S., Lee C.S. (2002). One-pot synthesis of 2*H*-pyrans by indium(iii) chloride-catalyzed reactions. Efficient synthesis of pyranocoumarins, pyranophenalenones, and pyranoquinolinones. Bull. Korean Chem. Soc..

[38] Jung E.J., Lee Y.R., Lee H.J. (2009). Iodine-catalyzed one-pot synthesis of 2*H*-pyrans by domino Knoevenagel/6π-electrocylization. Bull. Korean Chem. Soc..

[39] Burke M.D., Schreiber S.L. (2004). A planning strategy for diversity-oriented synthesis. Angew. Chem. Int. Ed..

[40] Leutbecher H., Williams L.A.D., Rösner H., Beifuss U. (2007). Efficient synthesis of substituted 7-methyl-2*H*,5*H*-pyrano[4,3-*b*]pyran-5-ones and evaluation of their in vitro antiproliferative/cytotoxic activities. Bioorg. Med. Chem. Lett..

[41] Peng W., Hirabaru T., Kawafuchi H., Inokuchi T. (2011). Substituent-controlled electrocyclization of 2,4-dienones: Synthesis of 2,3,6-trisubstituted 2*H*-pyran-5-carboxylates and their transformations. Eur. J. Org. Chem..

[42] Kumari P., Narayana C., Dubey S., Gupta A., Sagar R. (2018). Stereoselective synthesis of natural product inspired carbohydrate fused pyrano[3,2-*c*]quinolones as antiproliferative agents. Org. Biomol. Chem..

[43] Jung E.J., Park B.H., Lee Y.R. (2010). Environmentally benign, one-pot synthesis of pyrans by domino Knoevenagel/6π-electrocyclization in water and application to natural products. Green Chem..

[44] Narayan S., Muldoon J., Finn M.G., Fokin V.V., Kolb H.C., Sharpless K.B. (2005). “On water”: Unique reactivity of organic compounds in aqueous suspension. Angew. Chem. Int. Ed..

[45] Riviera M.J., Mischine M.P. (2013). Green one-pot synthesis of 2*H*-pyrans under solvent-free conditions catalyzed by ethylenediammonium diacetate. Synth. Commun..

[46] Peña J., Moro R.F., Basabe P., Marcos I.S., Díez D. (2012). Solvent free L-proline-catalysed domino Knoevenagel/6π-electrocyclization for the synthesis of highly functionalised 2*H*-pyrans. RSC Adv..

[47] Chang L., Plevová K., Thorimbert S., Dechoux L. (2017). Preparation of substituted 2*H*-pyrans via a cascade reaction from methyl coumalate and activated methylene nucleophiles. J. Org. Chem..

[48] Xie P., Yang J., Zheng J., Huang Y. (2014). Sequential catalyst phosphine/secondary amine promoted [1 + 4]/rearrangement domino reaction for the construction of (2*H*)-pyrans and 2-oxabicyclo[2,2,2]oct-5-ene skeletons. Eur. J. Org. Chem..

[49] Hu J., Dong W., Wu X.Y., Tong X. (2012). PPh_3_-catalyzed [3 + 3] annulations of 5-acetoxypenta-2,3-dienoate with 1C,3O-bisnucleophiles: Facile entry to stable monocyclic 2*H*-pyrans. Org. Lett..

[50] Menz H., Kirsch S.F. (2006). Synthesis of stable 2*H*-pyran-5-carboxylates via a catalyzed propargyl-Claisen rearrangement/oxa-6π electrocyclization strategy. Org. Lett..

[51] Tejedor D., Delgado-Hernández S., Diana-Rivero R., Díaz-Díaz A., García-Tellado F. (2019). A domino strategy for the synthesis of 2*H*-pyrans from propargyl vinyl ethers. Eur. J. Org. Chem..

[52] Tejedor D., Díaz-Díaz A., Diana-Rivero R., Delgado-Hernández S., García-Tellado F. (2018). Synthesis and utility of 2,2-dimethyl-2*H*-pyrans: Dienes for sequential Diels−Alder/Retro-Diels−Alder reactions. Org. Lett..

[53] Tejedor D., Delgado-Hernández S., Peyrac J., González-Platas J., García-Tellado F. (2017). Integrative pericyclic cascade: An atom economic, multi C-C bond-forming strategy for the construction of molecular complexity. Chem. Eur. J..

[54] Tejedor D., Cotos L., Márquez-Arce D., Odriozola-Gimeno M., Torrent-Sucarrat M., Cossío F.P., García-Tellado F. (2015). Microwave-assisted organocatalyzed rearrangement of propargyl vinyl ethers to salicylaldehyde derivatives: An experimental and theoretical study. Chem. Eur. J..

[55] Chong Q., Wang C., Wang D., Wang H., Wu F., Xin X., Wan B. (2015). DABCO-catalyzed synthesis of 3-bromo-/3-iodo-2*H*-pyrans from propargyl alcohols, dialkyl acetylene dicarboxylates, and *N*-bromo-/*N*-iodosuccinimides. Tetrahedron Lett..

[56] Yamamoto Y., Okude Y., Mori S., Shibuya M. (2017). Combined experimental and computational study on ruthenium(II)-catalyzed reactions of diynes with aldehydes and *N*,*N*-dimethylformamide. J. Org. Chem..

[57] Tambar U.K., Kano V., Zepernick J.F., Stoltz B.M. (2007). The development and scope of a versatile tandem Stille-oxa-electrocyclization reaction. Tetrahedron Lett..

[58] Tambar U.K., Kano T., Stoltz B.M. (2005). Progress toward the total synthesis of saudin: Development of a tandem Stille-oxa-electrocyclization reaction. Org. Lett..

[59] Li C., Johnson R.P., Porco J.A. (2003). Total synthesis of the quinone epoxide dimer (+)-torreyanic acid: Application of a biomimetic oxidation/electrocyclization/Diels-Alder dimerization cascade. J. Am. Chem. Soc..

[60] Shoji M., Yamaguchi J., Kakeya H., Osada H., Hayashi Y. (2002). Total synthesis of (+)-epoxyquinols A and B. Angew. Chem. Int. Ed..

[61] Li C., Lobkovsky E., Porco J.A. (2000). Total synthesis of (±)-torreyanic acid. J. Am. Chem. Soc..

